# Preserving the Past: An Early Interview Improves Delayed Event Memory in Children With Intellectual Disabilities

**DOI:** 10.1111/cdev.12364

**Published:** 2015-04-15

**Authors:** Deirdre A Brown, Charlie N Lewis, Michael E Lamb

**Affiliations:** Lancaster University; University of Cambridge

## Abstract

The influence of an early interview on children's (*N* = 194) later recall of an experienced event was examined in children with mild and moderate intellectual disabilities (CWID; 7–12 years) and typically developing (TD) children matched for chronological (7–12 years) or mental (4–9 years) age. Children previously interviewed were more informative, more accurate, and less suggestible. CWID (mild) recalled as much information as TD mental age matches, and were as accurate as TD chronological age matches. CWID (moderate) recalled less than TD mental age matches but were as accurate. Interviewers should elicit CWID's recall as early as possible and consider developmental level and severity of impairments when evaluating eyewitness testimony.

Children with intellectual disabilities (CWID) and other developmental disorders are a particularly vulnerable group of witnesses; they are at increased risk of maltreatment (e.g., Sullivan & Knutson, 1998, 2000; Vig & Kaminer, 2002), and yet are less likely than typically developing (TD) children to have their complaints investigated (e.g., Reiter, Bryen, & Shachar, 2007). Furthermore, the testimonial competency of CWID is doubted by professionals and potential jury members (Brown & Lewis, 2013; Henry, Ridley, Perry, & Crane, 2011). By evaluating whether the factors that have been shown to influence the accounts of TD children contribute to the recall of CWID in the same way, we can begin to understand better the cognitive capacities of CWID. In this study, we examined two issues comparing CWID to TD children: (a) the influence of an early interview on subsequent reporting, and (b) the nature of children's reports across repeated interviews.

CWID tend to have particular information-processing challenges (e.g., attention, verbal memory, speed of information processing, working memory, and executive function impairments; Henry, 2010; Henry & Gudjonsson, 2007), and communication difficulties (e.g., delayed language development; Pinborough-Zimmerman et al., 2007), which may in themselves influence how and what children encode, retrieve, and report about their experiences. Furthermore, it has often been noted that CWID may be more vulnerable to suggestion as a result of greater tendencies toward compliance and acquiescence with adults (e.g., Henry & Gudjonsson, 2007), both of which may lead to enhanced suggestibility (e.g., Bjorklund et al., 2000). Studying CWID offers another avenue for understanding the contribution of such information-processing abilities to eyewitness testimony. Such research might also inform evidence-based guidelines to support the practice of forensic interviewers who must elicit complete, reliable and accurate accounts from children about their experiences and help educate those who evaluate CWID's testimony in court (e.g., judges, jury members).

Studies of individual differences in TD children have not consistently shown an association between IQ and the amount and accuracy of children's recall (e.g., Brown & Pipe, 2003; Chae & Ceci, 2005), possibly reflecting both methodological variations and a constrained range of IQ scores within the samples studied (Chae & Ceci, 2005; McFarlane, Powell, & Dudgeon, 2002). Including children with significantly impaired levels of cognitive function may reveal how more marked differences in ability influence memory for events. Recent research has also suggested that maltreated children and CWID may share similar cognitive processing profiles (such as lower intellectual ability and deficits in executive function; e.g., Trickett, Noll, & Putnam, 2011). The need to understand the relation between cognitive impairment and the recall of experienced events is thus important, not only for elucidating how retrieval and reporting may differ in CWID and TD children, but also for indicating how to help maltreated CWID and TD children to recount their experiences.

## CWID and Eyewitness Testimony

Although not extensive, the research examining CWID's abilities as eyewitnesses has, in the main, demonstrated that children with mild levels of cognitive impairment tend to perform like TD counterparts at the same developmental level (Henry, Bettaney, & Carney, 2011). This is consistent with a developmental delay perspective on intellectual disability (ID; Zigler & Balla, 1982), which proposes that developmental progression is similar in CWID and TD children, although the rates and endpoints differ. On some indices CWID may even be equivalent to TD children of the same age. This supports an optimal model of development in ID (Burack & Zigler, 1990), which proposes that, at least for some aspects of cognitive function, CWID are indistinguishable from TD children. As with TD children, the quantity and quality of information CWID (mild) provide consistently varies depending on how they are questioned (e.g., Agnew & Powell, 2004; Brown, Lewis, Lamb, & Stephens, 2012; Henry & Gudjonsson, 2003, 2007; Michel, Gordon, Ornstein, & Simpson, 2000), despite differences across studies in sample selection, comparison groups, event type, and delay (Brown et al., 2012).

The picture is somewhat dissimilar for children with more severe levels of cognitive impairment, however. Consistent with a difference model of ID (Ellis, 1969), these children tend to perform more poorly on most indices of eyewitness competency than TD children matched for developmental level (Brown et al., 2012; Henry & Gudjonsson, 2003). The difference model proposes that ID is characterized by more than simple disparities in rate and asymptotes of learning and that cognitive development is qualitatively different in CWID, with cumulatively increasing deficits relative to TD children.

Ideally, child victims of abuse make prompt disclosures of their experiences so that formal investigations can be rapidly initiated and concluded. In practice, however, the dynamics associated with disclosure (see Pipe, Lamb, Orbach, & Cederborg, 2007), and systemic processes within child protection, law enforcement, and judicial agencies ensure that considerable time often elapses before children are questioned about their experiences. As a result, researchers have examined the effects of delay on children's recall. As with TD children, CWID's accounts of their experience are less detailed (although not necessarily less accurate) after delays (e.g., Brown et al., 2012; Henry & Gudjonsson, 2003; Michel et al., 2000).

Further delays often occur before children are questioned or cross-examined in court. Delays between initial referral and courtroom testimony reportedly range from approximately 11 (United Kingdom; Plotnikoff & Woolfson, 1995) or 15 (New Zealand; Hanna, Davies, Henderson, Crothers, & Rotherham, 2010) months, to up to 2 years (United States; Quas & Sumaroka, 2011). It is important, therefore, to ask what happens to children's recall of their experiences over extended periods of time.

## Early Interviews Mitigate the Effects of Delay on Children's Subsequent Reports

Research with TD children suggests that an early interview, especially one that elicits a relatively complete or comprehensive account, may reactivate memories of the original experience and strengthen memory over time (Pipe, Sutherland, Webster, Jones, & La Rooy, 2004; Salmon & Pipe, 2000), especially if some forgetting has already occurred. Early interviews may also mitigate or inoculate against subsequent forgetting and preserve unique details of the children's experiences. Well-conducted interviews may increase the coherence of children's original accounts, leading to stronger event representation in memory, and enhance later retrieval. Early interviews may also improve children's resistance to subsequent misleading suggestions and reduce false reports (e.g., Quas et al., 2007). Studies examining the effects of early interviews on later recall have produced inconsistent findings, however, about whether there are benefits that may reflect differences in delays before the initial interview (e.g., Pipe et al., 2004), when the repeated interviews occur (Pipe et al., 2004; Salmon & Pipe, 2000), and concerning the ways in which memory is assessed (Peterson, 2011). In this study, we compared accounts provided after a 6-month delay by CWID who were and were not first interviewed soon after the target event.

## Repeated Interviews

A further aim of the study was to examine the nature of CWID's recall across two interviews. Repeated interviews frequently occur when maltreatment is being investigated (La Rooy, Lamb, & Pipe, 2009; Goodman & Quas, 2008). Children may be interviewed on more than one occasion when they appear reluctant to disclose but other investigative leads indicate that maltreatment probably occurred, when there are multiple events to be recounted, or when children have become fatigued or distressed during the initial interview. Investigators may presume that children with developmental disorders would particularly benefit from multiple interviews to mitigate attention deficits and other cognitive impairments (Michel et al., 2000). Children may be reinterviewed after longer delays when new information comes to light, when cases are reopened, as a refresher prior to a court appearance, and during the trial process itself (e.g., during direct, cross-, and reexamination; La Rooy, Katz, Malloy, & Lamb, 2010).

Repeated interviewing may lead to reminiscence (recollection of new, previously unreported information) or hypermnesia (an increase in the total amount of information reported relative to earlier retrieval attempts; Erdelyi, 1996). The act of retrieving and reporting information may also reactivate the original recall, consolidating the strength of memory traces and increasing subsequent access to retrieval cues (Rovee-Collier, Greco-Vigorito, & Hayne, 1993). With strengthened memory traces, children may be more likely to generate their own retrieval cues, which, in turn, may prompt additional information.

However, repeated interviewing may also compromise the reliability of children's accounts if errors reported during earlier interviews (either self-generated or in response to suggestive questioning) become incorporated into memory representations (e.g., Ceci, Huffman, Smith, & Loftus, 1994). Additionally, children may be exposed between interviews to information from other sources that is subsequently incorporated into their accounts (via source monitoring errors or social pressure). Children may also produce inconsistent accounts across interviews, reflecting errors of both omission and commission, in repeated retrieval attempts (Peterson, 2011). Such inconsistencies may affect credibility negatively in the eyes of investigators, lawyers, judges, and jury members.

The negative effects of repeated interviewing have typically been demonstrated in the context of suggestibility rather than when interviews follow recognized evidence-based practice (e.g., Goodman & Quas, 2008; Peterson, 2011). Studies that examine repeated recall using optimal interviewing techniques (i.e., open-ended questioning) show that, as with other dimensions of eyewitness testimony, effects reflect the delay and the questioning strategy employed (e.g., Goodman & Quas, 2008; La Rooy et al., 2009; Pipe et al., 2004). With short delays, reporting of increased amounts of information that is highly accurate is facilitated (i.e., both reminiscence and hypermnesia: La Rooy, Pipe, & Murray, 2005; Quas et al., 2007). When the delay between interviews is more substantial (6 months or more), new information reported is often inaccurate, whereas information that is consistently reported tends to be accurate (e.g., La Rooy et al., 2005; Salmon & Pipe, 2000).

## Research With CWID and Repeated Interviews

We identified three studies of CWID who were interviewed twice. Gordon, Jens, Hollings, and Watson (1994) examined recall of a series of performed or imagined events, immediately and again 6 weeks later, by 10-year-old CWID and TD children matched for mental age (MA). In the delayed interviews all children were less informative and less accurate. Consistent with a delay model of ID, children in both groups showed similar free recall, but the TD children were more accurate when recognition questions were asked, supporting a difference model.

Michel et al. (2000) examined recall of a simulated medical checkup, immediately and again 6 weeks later, by 10-year-old CWID and TD children matched for both chronological age (CA) and MA. Again, all children recalled less after a delay, and CWID performed like those matched for MA, consistent with a delay model of ID. In both studies, the content of children's reports across the two interviews (e.g., for consistency or reminiscence) were not compared, nor did they include children who were interviewed for the first time after 6 weeks, so the effects of the immediate interviews on later accounts could not be determined.

Finally, Henry and Gudjonsson (2003) examined recall of a witnessed interaction by 11- to 12-year-old CWID with either mild or moderate levels of cognitive impairment and TD children matched for both CA and MA, 1 day and 2 weeks after the event. All children reported more information during free recall in the second interviews, leading to increased total recall (hypermnesia). Children with mild levels of ID were similar CA matches in free recall, supporting an optimal model of ID, but were less responsive to cued recall questions than CA matches (but not MA matches) consistent with a delay model of ID. Suggestibility increased during the second interviews and children with ID were more likely to respond inconsistently to closed questions than both groups of TD children, supporting a difference model of ID. This study was the first to examine consistency across interviews in CWID, although it did not examine the accuracy of the children's new and consistent responses, and the effects of delay could not be separated from the effects of second retrieval attempts because no children were interviewed only once.

## The Current Study

The above studies shed some light on the ways in which CWID may compare with TD children. The studies differ, however, with respect to factors (the samples, stimulus events, and length of delay) that may contribute to variability in recall, and the severity of cognitive impairment has not been explored systematically. The degree of personal participation in the events also varied. Laboratory-based memory tasks using minimal exposure to witnessed stimuli (e.g., video clips) have questionable implications for our understanding of memory for meaningful personal experiences, such as instances of abuse. Personally experienced events are typically better recalled and described than those that have been witnessed (see, e.g., Murachver, Pipe, Gordon, Owens, & Fivush, 1996), and so we were interested in whether conclusions drawn from extant research using more constrained stimuli (e.g., witnessed events or discrete actions) would be supported when CWID were questioned about extended personal experiences.

We extended the ecological validity of previous research with CWID in several ways. We examined CWID's recall when delays were similar to those likely to be encountered in real-world settings, and included younger CWID and those with more severe IDs than in earlier studies, thereby extending the population to which findings can be applied. We expected on the basis of previous research that all children would benefit from early interviews when recounting their experiences several months later. We anticipated that children in the TD group matched for CA would provide the most detailed and accurate accounts and that children in the CWID (mild) group and the TD children matched for MA would report equivalently detailed and accurate accounts (delay model), whereas children in the CWID (moderate) group would report fewer details, and be less accurate (difference model) than all other groups. We also explored the content of children's reports across repeated interviews, to evaluate the consistency of what they reported. We expected that all children would report new information (reminiscence) during the second interview, but that the accuracy of this information would be lower than information that was consistently included in their accounts.

We studied the capacity of CWID to recall an experience under optimal interviewing conditions accurately by using the National Institute of Child Health and Human Development (NICHD) Investigative Interview Protocol (Brown et al., 2013; Lamb, Hershkowitz, Orbach, & Esplin, 2008). It is important that studies that may inform criminal proceedings involving CWID approximate as closely as possible the ways in which they may be interviewed in forensic settings. Using a modified version of the NICHD Investigative Interview Protocol thus allowed us to determine how well CWID could respond to a flexible and child-centered, exhaustive interviewing protocol, rather than the more constrained script typically used in laboratory-based studies.

Studies of CWID and eyewitness testimony have shown variability in performance according to how memory was assessed (e.g., free recall vs. responses to cued recall or recognition questions), and the nature of the memory task (e.g., accurate recall vs. response to suggestive questions). We therefore also included an assessment of suggestibility in our study by including a series of highly leading and misleading questions similar to those that might be encountered during cross-examination (Zajac, O'Neill, & Hayne, 2012). Over time children's recall may become more fragmented as memory traces decay, thereby leading children to be more susceptible to suggestive questioning. Previous exposure to misinformation through suggestive questioning may also influence subsequent reporting even in free recall (but see Quas et al., 2007). We anticipated that children in the TD group matched for CA would be the least suggestible and that children in the CWID (mild) and TD matched for MA groups would show similar levels of suggestibility, whereas children in the CWID (moderate) group would be most suggestible. We did not expect children who had been exposed to misinformation via the suggestive questions in an earlier interview to be any less accurate during the second interview, given the delay between the two interviews, and the brevity of their exposure to misinformation.

## Method

### Participants

Children (*N* = 196; 79 female and 117 male) were recruited from nine schools (four mainstream and five for CWID) in the Northwest of England. Approximately half of the sample were interviewed for the first time 6 months after the event (*n* = 94) and the remainder (*n* = 102) were first interviewed 1 week after the event. Ten of the participants who had been interviewed 1 week following the event could not be contacted for their second follow-up interview (CWID [moderate] *n* = 1, CA *n* = 8, MA *n* = 1), so only 92 were interviewed both 1 week and 6 months after the event. Descriptive characteristics of the sample can be found in Table 1.

**Table 1:** Characteristics of the Sample (Collapsed Across Children Interviewed First at 1 Week or 6 Months)

	CWID (moderate)	CWID (mild)	CA matches	MA matches
*N* (first interview at 6 months)	14	23	23	30
*N* (second interview at 6 months)	20	23	29	34
*n* (male)	23	30	26	38
*n* (female)	11	16	26	26
Mean age in months (*SD*)	117.71 (12.88)	116.87 (15.60)	112.35 (14.71)	75.47 (16.22)
Age range in months	90–139	86–147	86–138	50–111
Mean estimated IQ score (*SD*)	48.03 (3.13)	67.67 (7.03)	101.62 (10.42)	100.88 (10.59)
Range of estimated IQ scores	44–53	55–78	84–125	85–124

*Note*. CWID = children with intellectual disabilities; CA = chronological age; MA = mental age.

#### Age

Children were between 4 and 12 years of age (7–12 years for the CWID children). A univariate analysis of age (months) demonstrated a significant main effect of condition, *F*(3, 188) = 99.64, *p* < .001, 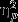 = .61, with Tukey tests (using the Tukey–Kramer adjustment here and below) indicating that children in the MA group were significantly younger than all others, who did not differ (all *p*s < .001). The mean age of the groups did not differ according to the timing of the first interview and no interaction between group and timing was evident.

#### Group Allocation

Children were categorized into four groups on the basis of their performance on four subtests (Picture Completion, Information, Block Design, and Vocabulary) of either the Wechsler Preschool and Primary Scale of Intelligence–Third edition, UK version (WPPSI–III UK; Wechsler, 2003) or the Wechsler Intelligence Scale for Children–Third Edition, UK version (WISC–III UK; Wechsler, 1992), and in the case of the ID groups, in conjunction with additional information reflecting adaptive function deficits or poor academic achievement consistent with a low level of intellectual function (as indicated by either attendance at a special school or targeted teaching assistance in mainstream schools). Children with estimated IQ scores of 55–78 were placed in the CWID (mild) group. Some of the children in this group (*n* = 9) had scores that were slightly above the guidelines of 70–75 outlined in the *DSM–IV–TR* (*Diagnostic and Statistical Manual of Mental Disorders,* 4th ed., Text Revision; American Psychiatric Association, 2000). Consistent with previous research (e.g., Agnew & Powell, 2004; Henry & Gudjonsson, 2003) these children were included in the mild intellectual impairment group, because the overall IQ scores were estimated and the children had well-documented cognitive and adaptive functional impairments. We conducted the analyses with these children and their associated MA and CA matches removed from the data set, with no difference in the pattern of findings, and so retained the full sample for analysis. Children were allocated to the CWID (moderate) group if their estimated IQ scores fell within the range 40–55. These participants were capable of basic verbal communication using at least phrase-based speech, confirmed in consultation with the children's teachers.

A number of a priori decisions were made to try and limit the contribution of comorbid conditions to children's performance, given that particular developmental disorders may be associated with specific cognitive function profiles (Henry, Bettaney, et al., 2011). Those with IDs associated with organic syndromes (e.g., Down syndrome) and those with diagnoses (confirmed or pending) of autistic spectrum disorder were excluded. Children were also excluded if they had comorbid conditions (e.g., attention deficit hyperactivity disorder, conduct disorder). Because theoretical models of ID focus on cultural-familial etiologies rather than ID arising from infections, trauma, or brain injuries (Zigler & Balla, 1982), we also planned to exclude any children with such histories, although in fact none were recruited. Children were included in the TD group if their estimated IQ scores fell within the average range. One child whose estimated IQ was 84 was included; this child was matched with a CWID whose score was 20 points lower. Univariate analysis of estimated IQ scores for the four groups revealed a significant main effect of condition, *F*(3, 188) = 375.80, *p* < .001, 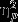 = .86; Tukey tests indicated that CWID (moderate) had lower scores than CWID (mild), who in turn differed from children in both of the TD groups (all *p*s < .001). Each group's estimated IQ scores did not differ according to the timing of the first interview.

#### Matching Samples

TD children were individually matched as closely as possible to CWID on the basis of gender and either CA or MA, to form two different control groups. Mental age was determined where possible from the tables provided in the Wechsler manuals (Wechsler, 1992, 2003). When MA estimates were not available from the Wechsler manuals, because the children's ages fell in the crossover band between the two instruments and the severity of ID made the range of MA estimates provided by the WISC–III UK discrepancy analysis tables insufficient, MA was estimated using the formula: IQ = (MA/CA) × 100 (Sattler, 2008).

### Procedure

#### Event

The event was class based and was typically conducted in either the children's classrooms or in the school hall and is described elsewhere (Brown et al., 2012). Each event was presented by research assistants and at least one member of the research team. Event durations ranged from 45 to 60 min. Children participated in three different activities (identifying hazards in pictures, learning how to care for a small cut, and tying a triangular bandage) in team groups. Partway through, an argument about using the equipment was staged by the event coordinator and another research assistant. At the end of the event, all children received a small gift (a novelty pencil). Deviations from the script were noted immediately afterward by the research assistants, and each station was also video recorded so that the accuracy of children's recall could be assessed.

#### Brief Cognitive Assessments

All cognitive assessments took place in a quiet room at the school during the week following the event (range = 3–7 days). Some children took part in the cognitive assessment session after they had been interviewed. At the end of the session, children were given a (different) small novelty gift in appreciation of their efforts.

#### Interview

The interviews were also conducted at school. Some children had been interviewed (by the same interviewer) 6 months earlier (1 week after the event). The same research assistant who conducted the cognitive assessment acted as interviewer to enhance rapport, with the other acting as a monitor. The monitor observed each interview and advised the interviewer during a break about how to clarify any ambiguous responses or elicit unreported details in the final stage of the interview. Three research assistants conducted the interviews; no effect of interviewer on total amount of information reported was evident, *F*(2, 174) = 0.06. All interviewers had a minimum of a masters in psychology, and completed a 2-day training workshop in the use of the NICHD Interview Protocol. The interviewers had completed several training interviews with children recruited as part of a separate study. Interviews were regularly monitored by the first author to ensure adherence to the Protocol, interviewers participated in feedback sessions that included viewing the videotapes of their interviews and reviewing the transcripts from them, and refresher training and feedback sessions were scheduled throughout the study. Both research assistants were present for each interview and provided additional feedback to each other after each interview to assist in maintaining fidelity and comparable performance.

Each interview began with rapport building using open-ended questions, typically about recent significant events (e.g., birthdays, holidays). The interview proper began with explanation of the “ground rules” (the importance of telling the truth, alerting the interviewer if they did not understand a question, the acceptability of “don't know” responses, and the need to correct the interviewer if she made a mistake). Each of these rules was accompanied by an example and an opportunity for the child to practice each rule. This was followed by practice in episodic memory recall, using what the child had done that day as the focus of the narrative. Focus was shifted to the staged event using a series of progressively informative prompts to help orient the children to the event the interviewers wished them to talk about (see Table 2).

**Table 2:** Prompts Used to Elicit Children's Initial Accounts of the Staged Event

1. I heard that a few weeks ago some people came to talk to your class about safety. I wasn't there but I'd like to know all about what happened. Tell me everything that happened from the beginning to the end.
2. I heard that you and your class went to (location of event) and learned about safety. Tell me all about what happened.
3. I heard that after they talked about safety they gave you a ______. Tell me all about what happened.
4. I heard you learned about looking after a cut, and got a sticking plaster. Tell me all about what happened.

The interview progressed using the prompts and structure outlined in the NICHD Protocol (Brown et al., 2013; Lamb et al., 2008). After the most *open invitations* (e.g., “Tell me about that time”) were used, children were encouraged to report as much as they could recall using a variety of different prompts. Information reported by the children was used to form *cued invitations* (e.g., “You mentioned you got to choose a plaster; tell me more about choosing the plaster”). Children were also asked *direct* (specific “wh-”) *questions* if needed to clarify unclear or contradictory aspects of their reports (e.g., “Which plaster did you choose?”). *Option-posing questions* asked children to choose from an array of interviewer-provided options (e.g., “Did you or your partner go first?”) or required a yes–no response. Direct and option-posing questions were followed up or “paired” with open prompts (e.g., “Tell me more about that”). The Protocol has a flexible structure and so the use of different prompts and the progress of each interview varied. Irrespective of group, when children indicated that they could not recall anything further or were nonresponsive to requests for further information, the interviewer took a short break and consulted the monitor.

At the end of the recall interview, when the children indicated they could not recall anything further, they were asked a final series of suggestive questions. Some questions asked about events or details that did not occur (i.e., they were *misleading*) and some asked about things that had occurred (i.e., they were *leading*). Questions also varied depending on whether they were *closed*, requiring a yes or no answer (e.g., “Were you in the blue group?”), or *open*, requiring the children to provide the response (e.g., “What color was the group you were in?”). All children received the same number of questions, and the order of topic administration was held constant to reduce the number of variants of each version of the suggestive question script. Following the suggestive questioning, the children were thanked for their efforts and given a small novelty gift (e.g., a notebook or a set of coloring pens). All interviews were transcribed verbatim from the digital video recordings. All interviewer or child utterances (including facilitative utterances such as “mmhmm” or “uh huh”) were transcribed. Behavioral responses (e.g., children demonstrating how to tie a triangular bandage) were described in full.

#### Coding

Two separate coding schemes were developed, one for the information reported during the NICHD Protocol interview, and one for responses to the suggestive questions. The lead coder was not blind regarding the group membership of each child (CWID vs. MA vs. CA); participants tended to be grouped by the school they attended and it was not possible to remove this detail from the transcripts. A subset (10%) of all of the interviews conducted (i.e., both single and repeated interviews) was recoded by a member of the research team who was blind to the group membership of the child to ensure that awareness of group membership had not affected how the interviews were coded, and the lead coder also recoded a subset of the interviews (10%) to check that coding remained consistent across the entire data set. The range of kappa values was 0.58–0.96, with a mean of 0.91, well above the 0.70 level described as “acceptable” by Bakeman and Gottman (1997).

Interviewer utterances were coded as open invitations, cued invitations, direct questions, option-posing questions, suggestive prompts, or facilitators. The number of each type of utterance was also tallied.

Because the event was both staged and recorded, allowing us to determine exactly what happened (cf. field studies where a record of the child's experiences is typically lacking), we adopted a checklist approach to coding. This may also have reduced the impact of any linguistic differences between the different groups. We identified 311 elements comprising the event (including its general structure, details of the activities, the people and the setting), derived from the event script and also coded, as elaborations, statements that the children made reflecting their individual experiences (e.g., “our group finished first”). An utterance could be scored in several categories (e.g., “I was in the green group” would score for indicating that there were groups, that one of them was green, and that the child was in the green group). Children's responses were coded in relation to the type of interviewer prompt that had elicited them and as correct, incorrect, or ambiguous (when it was unclear what the children were referring to, or if accuracy could not be ascertained using the available records) for each item on the checklist. Information that was repeated or was clearly off-topic was not analyzed.

For the children who were interviewed twice, information reported during the second (6-month) interview was coded as new information or old (repeated or consistent) information by comparing it to what was said during the first (1-week) interview. For information to be coded as old, it had to be reported consistently across the two interviews (e.g., correct at both time points). If children reported information that scored the same category code (e.g., that there were groups with colors as identifiers) but the quality of the information differed across the two interviews (e.g., correct at 1 week [by reporting three correct colors] but incorrect at 6 months [by reporting incorrect colors]) they had two details coded as repeated and correct (that there were groups and that the latter were color coded) and one detail coded as new and incorrect (wrong color).

## Results

### Statistical Design

A series of 4 (*group*: CWID [moderate], CWID [mild], MA matched, CA matched) × 2 (*number of interviews*: 1 vs. 2) factorial analyses of variance (ANOVAs) were conducted. When children's responses were examined in relation to the type of interviewer utterance, a third, within-subjects factor, *interviewer prompt*, with four levels (open invitations, cued invitations, direct questions, and option-posing questions) was added. Where data are reported as proportions, they were arcsin transformed (as recommended by Winer, 1970) and outliers were removed (even though neither action changed the pattern of results reported here, nor did rescoring the outliers so that they fell within the normal range) before analyses were conducted. If problems of sphericity were identified, Greenhouse–Geisser adjustments were made. These are identified by nonstandard degrees of freedom in the denominator. We present a relatively conservative effect size measure (partial eta-squared [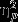]) to show the unique contribution of the relevant factor to the overall analysis. Tukey tests (*p* < .05) were conducted to unpack significant effects for group.

### How Do Previous Interviews and Children's ID Influence Reports?

Three measures examined different aspects of the children's reports (Table 3). First, we examined the number of prompts needed to get the children to recall the event. The Group × Number of Interviews ANOVA revealed a significant main effect of interview number, *F*(1, 188) = 20.82, *p* < .001, 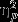 = .10; a significant main effect of group, *F*(3, 188) = 5.83, *p* = .001, 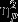 = .09; and a significant interaction, *F*(3, 188) = 3.07, *p* = .03, 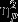 = .05. To investigate the interaction, simple effects analyses examined the effect of group separately for children who participated in one or two interviews (see Table 2). Univariate ANOVAs showed no significant main effect of group for children interviewed for the first time at 6 months, whereas a significant main effect of group was evident for children who were being interviewed for the second time, *F*(3, 98) = 7.76, *p* < .001, 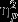 = .19. Tukey tests showed that children in the CA-matched group required a similar number of prompts as those in the CWID (mild) and MA-matched groups and fewer than CWID (moderate; all *p*s < .001), and that children in the CWID (mild) group required fewer prompts than children in both the CWID (moderate; *p* < .001) and MA groups (*p* = .033), who did not differ significantly from each other.

**Table 3:** Mean Scores (Standard Deviations) on Four Measures Assessing Children's Reports of Their Experiences

Interview number	CWID (moderate)	CWID (mild)	MA matched	CA matched	Total
Number of prompts					
First	3.57 (1.83)	3.43 (1.41)	2.97 (1.61)	2.52 (1.04)	3.06 (1.50)
Second	3.15 (1.63)	1.43 (0.79)	2.47 (1.66)	1.66 (1.08)	2.14 (1.47)
Total	3.32 (1.70)	2.43 (1.52)	2.73 (1.64)	2.04 (1.14)	2.58 (1.55)
Total information reported					
First	38.86 (31.19)	65.30 (26.63)	58.47 (30.62)	82.39 (27.02)	63.07 (31.60)
Second	48.05 (44.26)	79.48 (23.81)	70.27 (34.24)	95.59 (23.70)	75.19 (31.35)
Total	44.26 (26.01)	72.39 (25.98)	64.00 (32.65)	89.75 (25.83)	69.38 (31.96)
Accuracy of reports					
First	0.68 (0.14)	0.73 (0.09)	0.73 (0.13)	0.80 (0.07)	0.74 (0.11)
Second	0.75 (0.10)	0.81 (0.09)	0.79 (0.07)	0.83 (0.07)	0.80 (0.08)
Total	0.72 (0.12)	0.77 (0.10)	0.76 (0.11)	0.82 (0.07)	0.77 (0.10)
Accuracy of responses to scripted suggestive questions					
First	0.38 (0.16)	0.45 (0.16)	0.49 (0.19)	0.53 (0.08)	0.47 (0.16)
Second	0.36 (0.13)	0.52 (0.17)	0.51 (0.18)	0.64 (0.17)	0.52 (0.19)
Total	0.37 (0.14)	0.49 (0.17)	0.50 (0.18)	0.59 (0.14)	0.50 (0.18)

*Note*. CWID = children with intellectual disabilities; CA = chronological age; MA = mental age.

Second, we assessed the amounts of information reported. The Number of Interviews × Group ANOVA with number of details reported in the second interview as the dependent variable revealed a main effect of interview number, *F*(1, 188) = 8.60, *p* = .004, 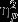 = .04, and group, *F*(3, 188) = 18.78, *p* < .001, 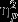 = .23, but no significant interaction. Consistent with our predictions, children who had previously been interviewed reported significantly more details than those interviewed for the first time at 6 months. CA-matched children reported more details than those in the other groups (for CWID [mild] *p* = .01, for MA and CWID [moderate] *p* < .001), and the CWID (moderate) children reported fewer than those in all other groups (all *p*s < .001, but there were no differences between the CWID (mild) children and the MA-matched controls.

We also examined the accuracy of children's statements (number of correct pieces of information as a proportion of the total amount of information provided in each interview). The Number of Interviews × Group ANOVA revealed effects for interview number, *F*(1, 187) = 19.44, *p* < .001, 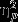 = .09, and group, *F*(3, 187) = 6.73, *p* < .001, 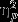 = .10, but no interaction. As expected, children were more accurate when they had been interviewed previously. In contrast to our predictions about recall accuracy, the children in the CWID (mild) group were not significantly different from that of children in any of the other groups. CA-matched children were more accurate than children in the MA-matched (*p* = .006) and CWID (moderate) groups (*p* < .001), who did not differ significantly.

### Which Interviewing Strategies Were Most Effective?

First we examined how many questions were posed during the interviews. The two-way ANOVAs revealed a significant main effect of group, *F*(3, 187) = 7.22, *p* < .001, 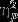 = .10, but no effect of interview number and no interaction. Children in the CA-matched group were asked fewer questions than children in either of the CWID groups, who did not differ. Children in the MA-matched group were asked similar numbers of questions as those in the CWID (mild) group but fewer than those in the CWID (moderate) group.

To explore the relations between the types of question asked and the nature of the children's responses, we first examined the relative proportions of information obtained in response to each of the four main types of prompts used by the interviewers: open invitations, cued invitations, direct questions, and option-posing prompts. Information reported in response to suggestive questions was not included because they were so infrequently used. Table 4 reports this information for each group at each time point. A Group (between) × Prompt Type (within) ANOVA revealed a significant main effect for prompt type, *F*(2.55, 477.62) = 52.22, *p* < .001, 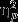 = .22, and a significant Group × Prompt Type interaction, *F*(7.66, 477.62) = 8.17, *p* < .001, 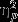 = .12.

**Table 4:** Proportions and Accuracy of Children's Responses (Standard Deviations) to the Four Main Types of Interview Prompts

	Proportion of information		Accuracy	
Group	First interview	Second interview	First interview	Second interview
Moderate CWID				
Open invitations	.15 (.10)	.16 (.13)	.95 (.08)	.91 (.10)
Cued invitations	.15 (.14)	.18 (.11)	.84 (.13)	.76 (.19)
Direct prompts	.27 (.17)	.39 (.16)	.68 (.14)	.67 (.25)
Option posing	.18 (.12)	.19 (.13)	.62 (.20)	.50 (.21)
Mild CWID				
Open invitations	.19 (.11)	.33 (.14)	.89 (.11)	.91 (.08)
Cued invitations	.30 (.12)	.26 (.11)	.78 (.16)	.81 (.12)
Direct prompts	.33 (.12)	.30 (.15)	.63 (.16)	.70 (.17)
Option posing	.13 (.08)	.11 (.07)	.53 (.21)	.62 (.20)
MA				
Open invitations	.17 (.13)	.23 (.16)	.92 (.10)	.90 (.11)
Cued invitations	.27 (.17)	.27 (.16)	.81 (.10)	.81 (.10)
Direct prompts	.33 (.18)	.34 (.12)	.65 (.16)	.72 (.11)
Option posing	.12 (.10)	.11 (0.11)	.63 (.24)	.50 (.21)
CA				
Open invitations	.30 (.14)	.33 (.14)	.88 (.10)	.93 (.07)
Cued invitations	.33 (.13)	.33 (.12)	.78 (.13)	.84 (.08)
Direct prompts	.26 (.11)	.25 (.12)	.69 (.20)	.73 (.14)
Option posing	.08 (.07)	.07 (.04)	.55 (.29)	.54 (.30)

*Note*. CWID = children with intellectual disabilities; CA = chronological age; MA = mental age.

To unpack the interaction, paired comparisons were conducted separately for each group (see Table 4). The proportion of information in the CWID (mild) group's reports that was elicited by invitations, cued that invitations and direct questions did not differ significantly, but less information was elicited using option-posing prompts (all *p*s < .001). Children in the CWID (moderate) group reported proportionally more details in response to direct questions than to invitations, cued invitations, and option-posing questions (all *p*s < .001), which did not elicit significantly different proportions of the overall information. Children in the CA-matched group reported proportionally more details in response to open invitations and cued invitations, which did not differ, than to direct (open invitations *p* = .048; cued invitations *p* = .008) or option-posing prompts (both *p*s < .001). For this group, direct questions elicited more details than option-posing questions (*p* < .001). Children in the MA-matched group reported the greatest proportion of information in response to direct questions (open invitations and option posing *p* < .001; cued invitations *p* = .044), followed by cued invitations, which in turn elicited more information than invitations (*p* = .010), which were superior to option-posing prompts (open invitations *p* < .001; cued invitations *p* = .003).

Table 4 shows the accuracy of the information provided by the children in response to the different types of interviewer prompt. The Group × Prompt Type ANOVA revealed a significant main effect of prompt type, *F*(2.52, 390.09) = 110.90, *p* < .001, 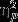 = .42. Paired comparisons showed that the statements made by children in each group were most accurate in response to open invitations and became less accurate as the questions became more focused and contained more interviewer input (all *p*s < .001, except direct question: option posing, where *p* = .002).

### Do a Previous Interview and Children's ID Affect Responses to Suggestive Questions?

A Number of Interviews × Group ANOVA revealed that children were significantly more accurate when responding to the suggestive questions at the end of their second interview if they had previously been interviewed, *F*(1, 186) = 4.54, *p* = .03, 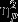 = .02. There was also a significant main effect for group, *F*(3, 186) = 11.67, *p* < .001, 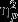 = .16. As expected, children in the CA group were the most (CWID [mild] *p* = .006; CWID [moderate] *p* < .001; MA *p* = .015) and children in the CWID (moderate) group were the least accurate (CWID [mild] *p* = .010; MA *p* = .001), with the children in the CWID (mild) and MA groups midway between those in the other two (Table 3).

### What Was the Quantity and Quality of Information Reported in Repeated Interviews?

We examined the amount and accuracy of information reported in repeated interviews by those children who were interviewed twice. A repeated measures ANOVA with the interview number (first vs. second) as the within-participant factor and group as the between-participant factor and number of details as the dependent variable showed a significant main effect of interview number, *F*(1, 97) = 27.34, *p* < .001, 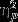 = .22; a significant main effect of group, *F*(3, 97) = 17.03, *p* < .001, 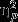 = .35; but no significant interaction. No evidence for hypermnesia was observed, with more information reported during the first interview (*M* = 89.11, *SD* = 34.72) than during the second (*M* = 75.21, *SD* = 31.48). Tukey tests to examine the effect of group showed that children in the CA-matched (*M* = 101.60, *SD* = 25.09) and CWID (mild) groups (*M* = 87.63, *SD* = 25.08) reported similar amounts of information, CWID (moderate) reported less information than all other groups (*M* = 50.20, *SD* = 25.09; all *ps* < .001), and children in the MA-matched group (*M* = 80.41, *SD* = 25.09) reported less than those in the CA-matched group (*p* = .009). A second repeated measures ANOVA examining the accuracy of children's accounts in the two interviews revealed significant main effects of interview number, *F*(1, 97) = 38.93, *p* < .001, 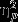 = .29, and group, *F*(3, 97) = 8.10, *p* < .001, 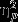 = .20, but there was no significant interaction. Children were more accurate during their first (*M* = 0.85, *SD* = 0.09) than their subsequent (*M* = 0.80, *SD* = 0.08) interview. Tukey tests to examine the effects of group showed that the CWID (moderate) group were less accurate (*M* = 0.76, *SD* = 0.09) than those in all other groups (CWID [mild] *p* = .006; CA *p* < .001; MA *p* = .039), who did not differ (CWID [mild] = .84, *SD* = 0.05; MA-matched = 0.82, *SD* = 0.05; CA-matched = 0.86, *SD* = 0.05).

### Did Repeated Interviews Yield New or Repeated Information?

We examined the nature of children's statements when they had been interviewed twice to compare the amount and accuracy of previously reported and new information (see Table 5). A repeated measures ANOVA with the type of information (repeated vs. new) as the within-participants factor, group as the between-participants factor, and number of details as the dependent variable showed a significant main effect of information type, *F*(1, 96) = 53.48, *p* < .001, 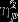 = .36; a significant main effect of group, *F*(3, 96) = 8.87, *p* < .001, 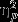 = .22; and a significant interaction, *F*(3, 96) = 894, *p* < .001, 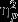 = .22. Repeated measures ANOVAs were conducted separately for each group to unpack the interaction. These showed significant effects of information type for the CA-matched, *F*(1, 27) = 55.66, *p* < .001, 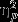 = .67; MA-matched, *F*(1, 28) = 11.62, *p* = .002, 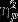 = .29; and mild CWID groups, *F*(1, 22) = 12.94, *p* = .002, 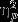 = .37. Consistent with our predictions in each group, children reported more repeated information than new, whereas, contrary to expectations, there was no significant difference between the amounts of new and repeated information reported by children in the moderate CWID group.

**Table 5:** Total Amount and Accuracy of Repeated and New Information (Standard Deviations) Reported in the 6-Month Interviews

	Amount of information		Accuracy	
Group	Repeated	New	Repeated	New
Moderate CWID	27.70 (18.37)	27.05 (13.32)	0.90 (0.14)	0.56 (0.17)
Mild CWID	48.09 (18.06)	31.43 (17.14)	0.92 (0.05)	0.62 (0.15)
MA	40.69 (26.21)	26.21 (12.65)	0.91 (0.13)	0.54 (0.16)
CA	63.79 (22.94)	30.43 (11.94)	0.94 (0.03)	0.61 (0.13)

*Note*. CWID = children with intellectual disabilities; CA = chronological age; MA = mental age.

A repeated measures Information Type (repeated vs. new) × Group ANOVA with accuracy as the dependent variable showed a significant main effect of information type, *F*(1, 95) = 444.44, *p* < .001, 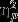 = .82, but no significant effect of group and no interaction. As predicted, information that was repeated in both interviews was more accurate than information newly reported at 6 months (see Table 5).

## Discussion

This was the first study to examine the effects of repeated interviewing over lengthy delays on the recall of experienced events in both TD children and CWID with varying degrees of disability. The findings reported above are consistent with but substantially enlarge upon previously reported findings. Specifically, we found, as others have done (Baker-Ward, Hess, & Flannagan, 1990; Pipe et al., 2004; Salmon & Pipe, 2000; Tizzard-Drover & Peterson, 2004), that children who were interviewed soon after an event recalled that event in greater detail and more accurately 6 months later than children interviewed for the first time. This was the first study to examine the content of CWID's reports across repeated interviews, and we showed that, as in research with TD children, participants who were interviewed twice reported new details when re interviewed, but these details were less reliable than those reported consistently (La Rooy et al., 2005; Salmon & Pipe, 2000). TD children's and those with mild ID's second interviews were composed of more repeated than new details, whereas CWID in the moderate range reported as much new as repeated information.

Our results suggest that for all children, regardless of cognitive ability, eyewitness testimony reflects a set of domain-specific abilities (e.g., retrieval of information during the interview vs. recognition and source monitoring during the suggestibility questions) rather than a more overarching set of general information-processing abilities. CWID in the mild range performed like CA matched counterparts on measures such as the number of prompts required to elicit initial recall in the repeated interview, and the accuracy of children's accounts (supporting the optimal performance model of ID; Burack & Zigler, 1990). In contrast, they were inferior to CA-matched TD children but equivalent to MA-matched TD children on measures such as the amount of information reported, and accuracy when responding to suggestive questions (supporting the delay model; Zigler & Balla, 1982). CWID in the moderate range consistently performed more poorly than both CA- and MA-matched TD children, supporting a difference model (Ellis, 1969). These mixed results are consistent with findings from laboratory-based assessments of CWID across different aspects of memory performance, showing variability according to the nature of the assessment, level of intellectual impairment, and nature (MA vs. CA) of the comparison groups (Weiss, Weisz, & Bromfield, 1986). Ours was the first study of episodic memory recall to suggest qualitative rather than quantitative differences between the capacities and limitations of CWID in the mild and moderate ranges.

Of course, given the heterogeneity that typically characterizes CWID, in terms of etiology of impairment (American Psychiatric Association, 2000), cognitive profiles (Henry, Bettaney, et al., 2011), specific information-processing difficulties (e.g., executive function, verbal memory; Henry, 2010), and other common comorbid deficits (e.g., language or communication difficulties; Pinborough-Zimmerman et al., 2007), even grouping on the basis of severity of ID may result in varying outcomes within a sample, which in part may account for variability across studies. As with TD children, we saw considerable variability within the CWID groups, indicating that cognitive function alone is not sufficient to account for recall performance. Our study provides further evidence of the complex interaction between when and how children are interviewed, child and event characteristics, and how performance is assessed on how well children can retrieve and describe their experiences (Peterson, 2011). An important direction for future research will be to explore psychosocial factors (e.g., socioeconomic status, parent–child interactions, temperament), and other aspects of cognitive and communicative function that have been associated with autobiographical memory, and that may be impaired in this population, to ascertain how they affect children's testimonial abilities. Such work increases our understanding of memory and narrative development in CWID, and also indicates how these children could be supported when they must communicate their experiences (e.g., forensic investigations, medical assessments, therapy).

Our findings are consistent with an emerging body of evidence demonstrating the ability of CWID, even those with moderate levels of cognitive impairment, to provide meaningful accounts of their experiences (Henry, Bettaney, et al., 2011), without a heavily scaffolded questioning approach. Both groups of CWID responded to broad, open-ended prompts that contained no interviewer input (i.e., invitations and cued invitations), providing between 30% and 34% (CWID [moderate]) and 49% and 59% (CWID [mild]) of their accounts in response to such prompts. The information they reported was highly accurate, even when they were interviewed for the first time after a considerable delay, and when they had previously been exposed to misinformation, whereas interviewer-led prompts elicited proportionally more erroneous information. These findings suggest that CWID can be valuable informants and witnesses when they have been abused. They underscore the need for investigators and courts to take their evidence more seriously than has been the case. The findings also underline the need for interviewers to give priority to very broad open-ended questions, and challenges notions that CWID must necessarily require more structured and specific prompting by interviewers. Both the youngest TD children and the more severely impaired CWID were most responsive to directive questioning. Although specific prompts can clearly be useful when interviewing both CWID and TD children (Hershkowitz, Lamb, Orbach, Katz, & Horowitz, 2012), the risks of eliciting inaccurate information (and the fact that children can report useful information in response to more open prompts) means that focused recognition questions should be delayed until the latter part of the interview.

The benefits of early interviewing for both CWID and TD children were evident on all of the variables we used. Having experienced earlier interviews, children subsequently re interviewed 6 months later required fewer introductory prompts to orient them to the target event. Not only did they report more information overall, but they required less cueing before beginning to do so. It is possible that, in addition to helping preserve their accounts, the early interview served as a reminder to the children of what they were there to talk about. The sociocultural theory of autobiographical memory development suggests that children learn how and what to remember and report when talking about past experiences from interactions with more experienced conversational partners (Nelson, 2013). Accordingly, the experience of interacting with an unfamiliar adult at school and talking in detail about a recent event may have served to model expectations for the subsequent interview, especially because it included practice narrating a recent episodic event. Indeed, the familiarity of the interviewer (and monitor) may have served as a context reinstatement cue (Bjorklund et al., 2000), although the effect of interviewer familiarity is not always salutary (e.g., Waterman, Blades, & Spencer, 2004). In the forensic setting, interviewers often face the challenge of getting children to begin providing their accounts without using leading questions that may compromise perceptions of those accounts. Thus, a further benefit of early interviewing may be that children learn their roles when interacting with interviewers and are therefore better able to access their memories without extensive initial prompting.

The number of additional details reported in a second interview, ranging from 10 to 14, was not insignificant. Although we did not examine the nature of these details (e.g., central vs. peripheral aspects of the event; cf. Peterson, 2011) we know that in forensic investigations, any additional relevant information may become a focus of further inquiry and provide the basis for further questioning. While our results suggest multiple interviews may yield more complete accounts without compromising reliability, it is also important that investigative needs do not compromise the well-being of maltreated children. For example, the potential benefits of eliciting further details must be weighed against the potential stress of experiencing an additional interview and recounting potentially distressing experiences. Although children do not always become distressed when talking about maltreatment (e.g., Sayfan, Mitchell, Goodman, Eisen, & Qin, 2008), many efforts have been made to limit the number of times that alleged victims are interviewed in order to minimize the stresses to which they are exposed (Quas et al., 2005).

Even though children who were interviewed twice had been exposed to misleading questioning in their initial interviews, their accounts were more accurate than those of children being interviewed for the first time after a delay. Goodman and Quas (2008) proposed that eliciting free narrative accounts of experiences soon after they occur may protect against subsequent suggestion, and our results support this. Other researchers have also demonstrated that in the absence of a biased interviewing style, exposure to misleading questions does not necessarily degrade accuracy in later interviews (Quas et al., 2007). These authors suggested that early exposure to misleading questions may have prepared children to be more resistant to suggestion by demonstrating that not all questions are answerable or aligned with what the children experienced. In our study, the delay between exposure to misinformation and the subsequent interview was much greater than in the Quas et al. (2007) study, which may also have diluted any potential effects of suggestive questioning. This is consistent with the theory that for many children, acquiescence to suggestive questions reflects compliance or psychosocial influences rather than enduring alterations of encoded representations (Bjorklund et al., 2000).

When examining the nature of children's reports across interviews, we saw the deleterious effects of delay on all aspects of recall (amount, accuracy, and suggestibility). Indeed, only a small fraction of the information potentially available to the children for reporting was included in their accounts, perhaps reflecting the fine-grained nature of our coding scheme. Errors of omission are common in studies of children's eyewitness testimony, in both TD and children with developmental disabilities (e.g., Bruck, London, Landa, & Goodman, 2007). Peterson (2011) has proposed that some of the interstudy variability in reported results (e.g., recall declining over time: Peterson, 1999; recall improving: Fivush, Sales, Goldberg, Bahrick, & Parker, 2004; no change with delay: Burgwyn-Bailes, Baker-Ward, Gordon, & Ornstein, 2001) may reflect the different dimensions on which children's recall has been evaluated or coded, as well as event characteristics (e.g., salience), and delay intervals. We tried to assess the completeness of the accounts (using a present vs. absent coding scheme for key features of the event) and we saw that the number of features children (CWID and TD) reported declined over time. By contrast, Peterson (2011), adopting the same approach, reported similar levels of recall over time. These divergent findings highlight the complex relation between delay and aspects of children's recall.

Some methodological limitations of the study need to be acknowledged. The children in our study experienced one long delay between the first and second interview. In forensic practice, children who are repeatedly interviewed are often required to recount their experiences in interviews repeatedly over several (possibly consecutive) days or weeks, and further research is needed to examine their effects. Like most studies of special populations, the sample size was also limited, so replication is essential. Although the event we studied was rich, novel, and interactive, it was pleasant; we cannot assume that the same competencies that would be observed had the experience been more stressful or traumatic. Studies suggest that, in general, children's memories of highly emotional negative experiences may be remarkably enduring (see Marche & Salmon, 2013, for a recent review). Events likely to precede court involvement tend to be physically and emotionally damaging, prolonged, or repeated, and may involve significant figures in the children's lives; all of these factors may themselves affect how and whether children disclose and the extent to which they are able to recall and report their experiences.

A further limitation of our event is the class-based nature of the experience—having children move through the event in groups made it difficult to determine whether individual children were attending to or engaged with the various activities. As such, group differences (e.g., reduced recall by younger TD children and both groups of CWID) may also reflect differences in the strength of encoding as well as in retrieval and reporting. We employed the NICHD Investigative Interview Protocol in our study to enhance the ecological validity of the research. Using a flexible protocol, however, limited our ability to make direct comparisons across the groups in terms of interviewing strategies and interviewer style that may in themselves have contributed to children's recall. This difficulty embodies in many ways the challenge of building a theoretical account of autobiographical memory, which must accommodate the range of factors associated with the child, the event under investigation, the interview process, the interviewer, and, importantly, the dynamic process that occurs between children and their interviewers, with each shaping both their own and their conversational partner's behavior (Gilstrap & Ceci, 2005).

These caveats notwithstanding, our study adds to the growing body of evidence documenting the ability of CWID to provide meaningful accounts of their experiences. To facilitate forensic participation, interviewers may need to assess children's developmental level and cognitive capacities (Brown et al., 2012; Henry, Bettaney, et al., 2011; Michel et al., 2000) to plan for developmentally sensitive interviewing. Although CWID in the moderate range may require more scaffolding from interviewers than those with milder degrees of impairment, it would be misguided to assume that they cannot benefit from the best practice approach advocated for TD children: an open-ended style of questioning with more focused questioning delayed until later in the interview. As with TD children, repeated interviews may help CWID to preserve their memories of salient experiences. We did not observe any detrimental effects of earlier interviewing, perhaps reflecting the quality of the initial interviewing. The positive effects of an early interview add further weight to the widespread recommendation that delays between experiences and interviews about them be minimized for *all* children, regardless of age or cognitive ability.
